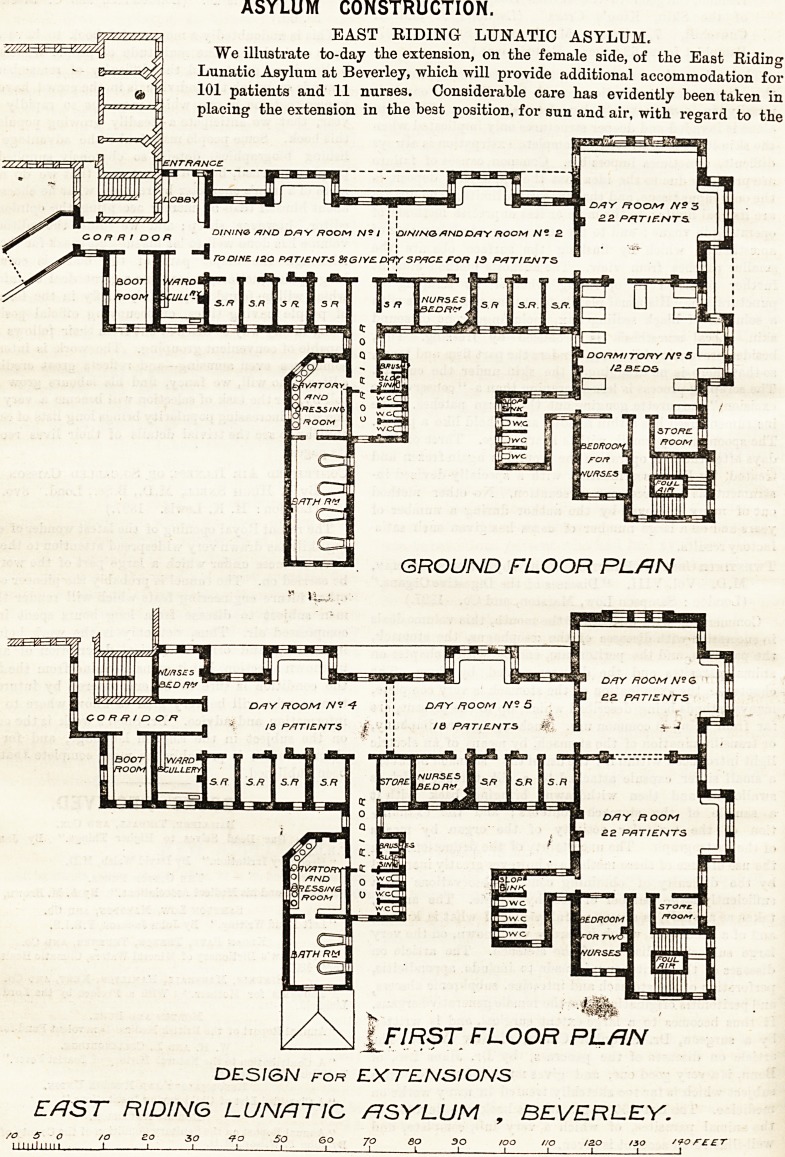# Asylum Construction

**Published:** 1897-06-12

**Authors:** 


					186 THE HOSPITAL. June 12, 1897.
The Institutional Workshop.
ASYLUM CONSTRUCTION.
EAST RIDING LUNATIC ASYLUM.
We illustrate to-day the extension, on the female side, of the East Riding
Lunatic Asylum at Beverley, which will provide additional accommodation for
101 patients and 11 nurses. Considerable care has evidently been taken in
placing the extension in the best position, for sun and air, with regard to the
ASYLUM CONSTRUCTION.
EAST RIDING LUNATIC ASYLUM.
We illustrate to-day the extension, on the female side, of the East Ridirig
Lunatic Asylum at Beverley, which will provide additional accommodation for
101 patients and 11 nurses. Considerable care has evidently been taken in
placing the extension in the beat position, for sun and air, with regard to the
ENTRANCE
FIRST FLOOR PLAN
DESI&N for EXTENSIONS
EAST RIDING LUNATIC ASYLUM , BEVERLEY.
/O 5 O
I I i 11111111 .
June 12, 1897. THE HOSPITAL. 187
?older buildings. The ground floor contains two dining
and day rooms facing nearly south, with large bays
giving light from the east and west, and a square
ay-room at the south-west angle, well lighted on
both sides. Behind the dining rooms are eight
single rooms for patients, and behind the day-room
is a domitory for 12 beds. There are two nurses'
bedrooms, and well-arranged lavatories, closets and
oath-rooms, "while a new corridor connects the ex-
tension with the administrative block. The first floor
is a repetition of the ground floor, but a day-room takes
the place of the dormitory. The second floor is de-
voted entirely to dormitories, which seem unduly
crowded, the beds being arranged in three rows, with a
floor space of only 51 square feet per bed. The central
row of beds can hardly have their proper allowance of
fresh air and light, and the arrangement is one which
should only be permitted when rendered necessary by
overwhelming considerations of economy. The architect
is Mr. C. H. Hebblethwaite.

				

## Figures and Tables

**Figure f1:**